# Immunometabolism and Sepsis: A Role for HIF?

**DOI:** 10.3389/fmolb.2019.00085

**Published:** 2019-09-06

**Authors:** Susan F. Fitzpatrick

**Affiliations:** School of Medicine and Medical Science, University College Dublin, Dublin, Ireland

**Keywords:** HIF, hypoxia inducible factor, metabolism, innate immune cells, sepsis, molecular mechanism

## Abstract

Metabolic reprogramming of innate immune cells occurs during both the hyperinflammatory and immunotolerant phases of sepsis. The hypoxia inducible factor (HIF) signaling pathway plays a vital role in regulating these metabolic changes. This review initially summarizes the HIF-driven changes in metabolic dynamics of innate immune cells in response to sepsis. The hyperinflammatory phase of sepsis is accompanied by a metabolic switch from oxidative phosphorylation to HIF-1α mediated glycolysis. Furthermore, HIF driven alterations in arginine metabolism also occur during this phase. This promotes sepsis pathophysiology and the development of clinical symptoms. These early metabolic changes are followed by a late immunotolerant phase, in which suppressed HIF signaling promotes a switch from aerobic glycolysis to fatty acid oxidation, with a subsequent anti-inflammatory response developing. Recently the molecular mechanisms controlling HIF activation during these early and late phases have begun to be elucidated. In the final part of this review the contribution of toll-like receptors, transcription factors, metabolic intermediates, kinases and reactive oxygen species, in governing the HIF-induced metabolic reprogramming of innate immune cells will be discussed. Importantly, understanding these regulatory mechanisms can lead to the development of novel diagnostic and therapeutic strategies targeting the HIF-dependent metabolic state of innate immune cells.

Sepsis is defined as life-threatening organ dysfunction caused by an uncontrolled host immune response to infection (Wentowski et al., 2019). Epidemiological assessment projects that there are 31.5 million case of sepsis annually worldwide, resulting in ~5.3 million deaths (Wentowski et al., 2019). Despite the frequency with which the disease occurs, diagnostic tests, and treatments for sepsis and septic shock remain limited.

Traditionally the acute phase of sepsis is classified as innate immune system activation resulting in a strong pro-inflammatory response, to try to eliminate the underlying pathogen. While, the late phase is characterized by an immuno-suppressed state producing an anti-inflammatory response to facilitate tissue repair (Van Wyngene et al., 2018). However, the pathogenesis of sepsis can only partially be explained by an aberrant inflammatory response. Recent research has shown that metabolic deregulation also plays a vital role in sepsis progression (Weis et al., 2017).

In healthy individuals, there is a balance between energy input and energy output. However, in septic patients, this energy balance is significantly altered. An initial hypermetabolic state consisting of increased oxygen consumption (Kreymann et al., 1993), elevated ATP production, hyperglycemia (Wernly et al., 2016) and protein, fat, and carbohydrate catabolism (reviewed Englert and Rogers, 2016), rapidly develops. A hypometabolic states will eventually occur with decreased oxygen consumption, mitochondrial respiration, and ATP production (Singer et al., 2004). It has been proposed that this hypometabolism allows cells to enter a hibernation like state that serves as a protective mechanism to aid recovery (Singer et al., 2004).

Immune cells contribute to the systemic changes in metabolism by altering their metabolic profiles in response to the immunological state i.e., pro- or anti-inflammatory, during sepsis (Kelly and O'Neill, 2015). This key adaptive feature controls immune cell function and fate and plays an important role in sepsis symptomology including; tachycardia, hypo-/hyper-thermia, tachypnea and hypotension and disease progression. Therapeutic modulation of immune cell metabolism could therefore potentially alter the inflammatory state during sepsis and thus improve patient prognosis.

In the first part of this review the metabolic changes which occur in innate immune cells during sepsis will be briefly discussed. In the second part, the role of the hypoxia inducible factor (HIF) pathway in mediating these metabolic changes and the molecular mechanisms controlling HIF signaling will be discussed.

## Innate Immune Cell Metabolism

The innate immune system is the first line of host defense against for example an invading pathogen. It consists of physical barriers including endothelial and epithelial cell layers and effector cells importantly; macrophages, monocytes, leukocytes, natural killer cells, mast cells, neutrophils, and dendritic cells (Turvey and Broide, 2010). Innate immune cells remain largely quiescent in the physiological state. In response to an invading pathogen the innate immune system stimulates an inflammatory response culminating in the clearance of a pathogen by phagocytosis and activation of the adaptive immune response. However, when a disproportionate innate immune response occurs excessive inflammation and organ failure allow sepsis and septic shock to develop. Recent advances in experimental models of sepsis and clinical sepsis have identified that numerous metabolic changes occur in innate immune cells, during sepsis both the acute hyperinflammatory phase, and the late immunotolerant phase. This metabolic remodeling appears to be vital for the activation and exertion of immune functions by these cells (Escoll and Buchrieser, 2018).

### Glycolysis

Glycolysis is an essential metabolic pathway through which cells generate energy in the form of ATP. Following uptake through the GLUT1 transporter, glucose is converted into pyruvate through a complex series of enzymatic reactions, generating a total of 2 ATP, and the products necessary to allow the TCA cycle and oxidative phosphorylation (OXPHOS) to occur. During sepsis a metabolic switch from OXPHOS to glycolysis occurs in innate immune cells including; macrophages, neutrophils, leukocytes, and dendritic cells (Krawczyk et al., 2010; Haschemi et al., 2012; Tannahill et al., 2013; Tavakoli et al., 2013; Everts et al., 2014; Freemerman et al., 2014; Cheng et al., 2016). While, the glycolytic inhibitor 2-deoxy-d-glucose (2-DG) dose dependently inhibits this switch, thereby inhibiting the release of early and late proinflammatory cytokines (Yang et al., 2014). Upregulation of pyruvate kinase M2 (PKM2) enables this metabolic switch in macrophages and administration of a specific PKM2 inhibitor, decreases glycolysis, and improves survival outcomes in a mouse model of intraabdominal sepsis (Yang et al., 2014). These studies show that the acute phase of sepsis characterized by a strong inflammatory response requires a corresponding activation of glycolytic pathways to drive the inflammatory response in macrophages and monocytes. Interestingly however, as monocytes enter an immunotolerant state their dependence on glycolysis decreases (Cheng et al., 2016).

In agreement with the studies on macrophages/monocytes early studies in animal models of sepsis also reported enhanced glycolysis in leukocytes (Haji-Michael et al., 1999). Subsequent studies have shown that during the initial hyperinflammatory phase genes encoding key enzymes associated with glycolysis are up-regulated in human peripheral blood leukocytes (Haimovich et al., 2010). In contrast, during the immunotolerant phase whole blood leukocytes under express genes involved in glycolysis (Cheng et al., 2016; van Vught et al., 2016).

### Pentose Phosphate Pathway (PPP)

The PPP, which occurs in parallel to glycolysis, involves the oxidation of glucose to generate NADPH, and carbohydrates for biosynthesis. It is further divided into an oxidative and non-oxidative branch. During oxidative PPP glucose 6-phosphate dehydrogenase (G6pd) converts glucose 6-phosphate into ribose 5-phosphate (R5P). The non-oxidative branch recycles R5P to glycolytic intermediates or to generate pentose phosphates. Upon LPS stimulation glucose flows through both the glycolytic pathway and the PPP, leading to significant increases in both oxidative and non-oxidative PPP activity in macrophages (Haschemi et al., 2012). Similar increases in PPP activity, have also been reported in leukocytes harvested from mice following cecal ligation and puncture (CLP) (Haji-Michael et al., 1999). On the other hand, it has also been reported that LPS induces a small decrease in PPP fluxes in macrophages (Rodriguez-Prados et al., 2010).

Macrophages with a G6pd mutation show decreased PPP activity and increased bacterial burden and worse survival outcomes in an animal model of sepsis (Wen et al., 2015). Furthermore, newborn infants born with a G6pd deficiency are more susceptible to sepsis due to a lack of leukocyte bactericidal activity (Abu-Osba et al., 1989). Similarly adults with leukocyte G6pd deficiency are also more susceptible to infection due to neutrophil (Mamlok et al., 1987) and monocyte (Spolarics et al., 2001) dysregulation. Although increased PPP metabolism in innate immune cells appears to be extremely important in controlling bactericidal burden during the acute phase of sepsis further studies are required to elucidate its role during the late stages.

### Tricyclic Acid Cycle (TCA)

The TCA cycle is the final common pathway in the metabolism of carbohydrates, fats, and amino acids and is central for connecting numerous individual metabolic pathways. The switch to glycolysis in macrophages during sepsis, coincides with increased accumulation of the TCA intermediates, fumerate, malate, and succinate (Tannahill et al., 2013; Mills et al., 2016). Despite the increase in these intermediates metabolic flux analysis showed that there is an overall decrease in TCA cycle activity (Tannahill et al., 2013). Moreover, in human peripheral blood mononuclear cells, acutely stimulated with *Candida albicans* or *Escherichia coli* derived LPS, the enhanced expression of mRNA encoding components of the glycolytic pathway was associated with a corresponding downregulation in mRNA encoding rate-limiting enzymes of the TCA cycle (Cheng et al., 2016). But TCA cycle intermediates are also produced by alternative sources. Indeed, in response to LPS macrophages increase succinate production through glutamine metabolism proceeding through both the GABA shunt and α-ketogluterate (Tannahill et al., 2013) and through mitochondrial oxidation of succinate via succinate dehydrogenase, driving ROS production (Mills et al., 2016). Timing appears to play a significant role in TCA up- or down-regulation in innate immune cells during sepsis. Exposure of dendritic cells to LPS for 1 h significantly increased citrate levels but failed to alter other TCA components including itaconate, fumarate, and malate. In contrast, after 3 h of LPS stimulation itoconate and fumarate were significantly downregulated, while citrate and malate remained unchanged (Everts et al., 2014). A greater understanding of the role of the TCA cycle and intermediates in innate immune cells during both the hyperinflammatory and immunotolerant phases of sepsis is needed, particularly in light of recent pre-clinical evidence showing that modulation of these intermediates is beneficial for sepsis therapy. For example, decreasing succinate levels *in vivo* using dimethyl malonate, a succinate oxidation inhibitor (Mills et al., 2016) or vigabatrin an inhibitor of the key GABA shunt enzyme GABA transaminase, is protective in mouse models of sepsis (Tannahill et al., 2013).

Citrate, another TCA intermediate, is also altered in innate immune cells during sepsis. It is formed from oxaloacetate and acetyl-CoA by the enzyme citrate synthase. In the presence of ATP, the enzyme ATP-citrate lyase (ACLY) converts citrate back to oxaloacetate and acetyl-CoA. LPS induces ACLY activation in macrophages contributing to increased prostaglandin synthesis, reactive oxygen species (ROS) and nitric oxide (NO) production (Infantino et al., 2013).

### Arginine Metabolism

Arginine is a precursor for a wide range of compounds and as a result is involved in multiple metabolic processes. Arginine metabolism can occur via two principle groups of enzymes, nitric oxide synthase (NOS) including inducible NOS (iNOS), neural NOS (nNOS), or endothelial NOS (eNOS) giving rise to NO and citrulline or through the arginase (Arg) enzymes, Arg1/2, producing ornithine, and urea. In response to LPS, macrophages drive iNOS expression but suppress Arg activity, thereby increasing NO levels (Takeda et al., 2010). NO in turn regulates processes involved in sepsis progression, including; vascular function and pathogen defense (Winkler et al., 2017). Intriguingly, global loss of the iNOS gene is associated with increased mortality of septic mice (Cobb et al., 1999). Furthermore, the loss of neutrophil iNOS does not alter the course of sepsis progression (Wang et al., 2012). Alterations in the iNOS Arg balance within innate immune cells may therefore be insufficient to confer a positive outcome in septic patients. Indeed even though LPS administration to Arg-1 deficient macrophages leads to increased NO (El Kasmi et al., 2008; Wijnands et al., 2014) overall survival was comparable to controls (El Kasmi et al., 2008).

Intriguingly, in addition to its role in regulating arginine metabolism iNOS has also been linked to glycolysis regulation. Indeed, LPS induced TLR4 stimulation causes the activation of iNOS and in turn NO, which inhibits the electron transport chain. As a consequence, activated dendritic cells enhance their glycolytic rates to prevent against bioenergetic collapse (Everts et al., 2012). Further studies have shown that in response to LPS dendritic cells have an early iNOS-independent and a late iNOS-dependent control on increases glycolysis (Everts et al., 2014).

There are additional levels of regulation for the enzymes involved in arginine metabolism. For example, NOS is inhibited by asymmetric dimethylarginine (ADMA) in a competitive manner. ADMA levels are in turn controlled by dimethylarginase-dimethylalaminohydrolase-1 and 2 (DDAH1 and 2) activity, that inactivate ADMA by cleavage. DDAH2 is predominantly expressed in immune cells. Intriguingly, significantly lower levels of DDAH2 were found in the PBMC of septic patients and correlated with a more severe disease (Winkler et al., 2017). In agreement, with these observations a monocyte specific deletion of DDAH2 resulted in impaired NO production from L-arginine and a higher incidence of severe illness (Lambden et al., 2015). Therefore, these studies suggest immune cell NO has an important role in pathogen defense.

### Fatty Acid β Oxidation

Fatty acid β oxidation is a second major source of fuel for cells. It is a complex catabolic process during which long chain fatty acids are converted into acetyl-CoA, for the TCA cycle and NADH and FADH_2_ for oxidative phosphorylation. Di- and tri-acylglycerols indicating increased fatty acid synthesis has been reported in LPS activated macrophages (Tannahill et al., 2013). However, the activation of fatty acid oxidation in innate immune cells during sepsis is a complex time dependent process. During the acute phase of sepsis response in monocytes and leukocytes the dominant metabolic profile is glycolysis with decreased expression of genes related to fatty acid oxidation. However, when glycolysis is impaired during the immunotolerant phase there is a switch to increased fatty acid oxidation (Liu et al., 2015). A down-regulation in glycolysis would be predicted to be accompanied by increased fatty acid synthesis, in order to maintain the demanding energy requirements of the immune cells. Unexpectedly one study reported that monocytes and leukocytes rendered tolerant or isolated from septic patients have decreased levels of fatty acid transporters (Cheng et al., 2016). An alternative theory to a switch from glycolysis to fatty acid β oxidation is that glycolysis regulates fatty acid synthesis. Indeed, in dendritic cells treated with LPS glycolysis is required for the *de novo* synthesis of fatty acids by generating NADPH through the PPP and by replenishing the intermediates of the TCA cycle which are bring extracted for the synthesis of fatty acids (Everts et al., 2014). While more recently, it has been shown that when glucose supply is limited, monocytes undergo a metabolic shift toward oxidative phosphorylation, fueled largely by fatty acid oxidation at the expense of lipid droplets (Raulien et al., 2017).

Targeting fatty acid oxidation in immune cells is proving to be therapeutically useful in septic models. Lysophosphatidylcholine (LPC) is the major component of oxidized low-density lipoprotein and is markedly decreased in septic patients (Drobnik et al., 2003). LPC has several immunomodulatory functions including the activation of macrophages, monocytes and T lymphocytes (Murch et al., 2009). Treatment of mice with LPC reduces mortality in septic mice due to reduced neutrophil deactivation (Yan et al., 2004). Moreover, in macrophages and monocytes, LPC inhibits the release of the high-mobility group box 1 (HMGB1) (Chen et al., 2005), improving organ injury and dysfunction and inflammatory cytokine release caused by LPS and Gram-positive shock (Murch et al., 2009). Conversely, other studies have reported that inhibition of fatty acid metabolism maybe a more effective approach to sepsis treatment. Indeed, mice with a targeted deletion of mitochondrial uncoupling protein-2 (UPC-2) in their macrophages had inhibited lipid synthesis due to down regulation of fatty acid synthase, a key regulator of fatty acid synthesis. This resulted in improved survival in a mouse model of polymcirobial sepsis (Moon et al., 2015). Moreover, UPC-2 expression is overexpressed in human sepsis (Moon et al., 2015).

While, current data clearly supports changes in innate immune cell metabolism during sepsis, the molecular mechanisms governing these changes remain to be elucidated. One pathway which has gained significant importance in recent years is the HIF signaling pathway. In the final part of this review the involvement of HIF signaling in regulating metabolic changes in innate immune cells during sepsis will be discussed.

## HIF

**Figure 1:**
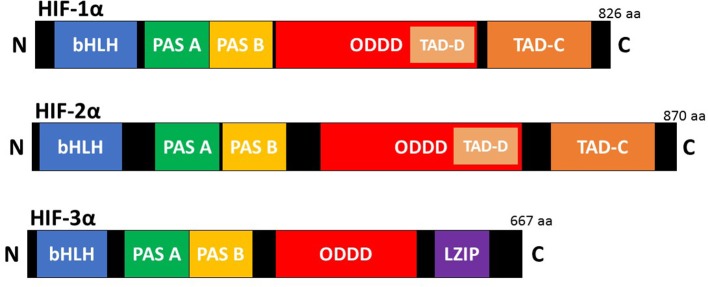
Schematic illustration of the domain structure of the HIF-α members. HIF-α consists of a bHLH (basic helix loop helix) and PAS (Per-ARNT-Sim homology) domain in the NH2-terminal, which are necessary for heterodimerization and DNA binding to the hypoxia response elements (HRE). Two transactivation domain(s) (TAD), which stimulate transcription, are present in the COOH-terminal of HIF-1α, and HIF-2α. TAD-C interacts with coactivators such as CBP/p300 to activate gene transcription. HIF-3α has a leucine zipper (LZIP) domain but lacks the TAD-C domain present in HIF-1α and HIF-2α.HIF-α also contains an oxygen-dependent-degradation (ODD) domain, which contains the conserved proline(s) hydroxylated by PHD and FIH to promote proteasomal degradation.

HIFs are the master regulators of the adaptive response to hypoxia (low oxygen). They are heterodimeric transcription factors composed of an oxygen dependent α subunit and a constitutively expressed β subunit [also known as aryl hydrocarbon receptor nuclear translocator (Arnt)]. Each subunit is a member of the basic helix-loop-helix/Per-Arnt-Sim homology (bHLH/PAS) family (Kaelin and Ratcliffe, 2008). The mammalian genome encodes three isoforms of the α subunit (HIF-1α, HIF-2α, and HIF-3α) and three paralogues of the β subunit (Arnt1, Arnt2, and Arnt3) have been identified (Lisy and Peet, 2008). HIF-1α is the most ubiquitously expressed isoform and shares 48% amino acid sequence homology with HIF-2α. It has a similar protein structure to both HIF-2α and HIF-3α (Figure 1). However, HIF-1α and HIF-2α have unique tissue expression profiles, embryonic deletion phenotypes, target genes, and physiological effects (Patel and Simon, 2008). Furthermore, it appears that HIF-1α has a key role in the acute response to hypoxia, while HIF-2α drives the response during chronic hypoxia. Compared to HIF-1α and HIF-2α, HIF-3α is less well characterized. This is due in part to the existence of multiple HIF-3α variants. Evidence shows that the full-length HIF-3α protein functions as an oxygen-regulated transcription activator and that it activates a unique transcriptional program in response to hypoxia (Duan, 2016). However, some short HIF-3α variants act as dominant-negative regulators of the action of HIF-1α and HIF-2α, while other HIF-3α variants can inhibit HIF-1α or HIF-2α by competing for the common HIF-β (Duan, 2016).

### HIF Regulation

There are two types of pathways involved in the regulation of HIF-α, oxygen dependent, and oxygen independent pathways.

#### Oxygen Dependent

**Figure 2:**
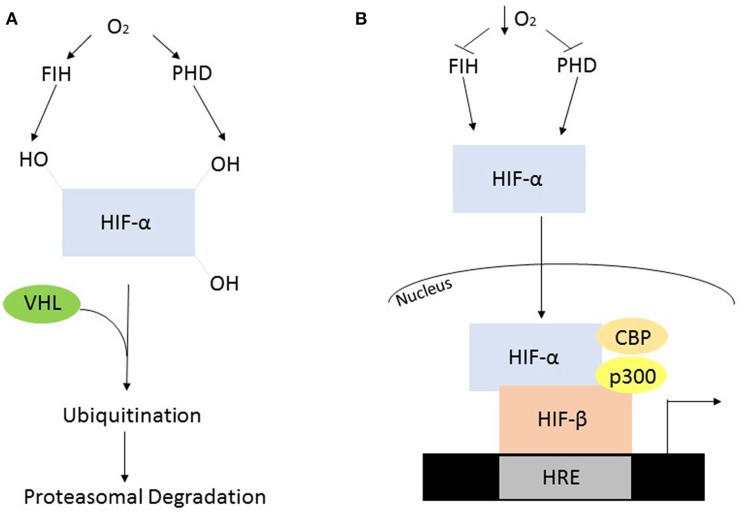
Oxygen dependent regulation of HIF-α. **(A)** In the presence of oxygen, the PHD's and FIH are active and hydroxylate HIF-α, constituting a recognition signal for the von Hipple Lindau (VHL), and subsequent ubiquitination and proteasomal degradation. **(B)** The absence of oxygen causes suppressed enzyme activity resulting in HIF-α stabilization, allowing it to form a heterodimer with HIF-β. This heterodimer will subsequently interact with various co-factors to initiate transcription.

In the presence of oxygen the HIF-α subunits are synthesized at a high rate but are rapidly degraded via post-translational modification by oxygen-dependent prolyl hydroxylase (PHD) enzymes. In humans, the PHDs hydroxylate two conserved proline residues, Pro402 and Pro564, which are present in the oxygen dependent domain of the α subunit (Huang et al., 2002). The mammalian genome encodes three PHD enzymes termed PHD1, 2, and 3. PHD2 is the predominant isoform contributing to the regulation of HIF-1α, while PHD1, and of HIF-2α (Webb et al., 2009). The post-translational hydroxylation allows an E3 ubiquitin ligase known as the von Hipple-Lindau to bind and target the HIF-α subunit for degradation by the 26S proteasome (Kaelin and Ratcliffe, 2008). While PHD-mediated proline modification regulates the stability of HIF-α the asparginyl hydroxylase, factor inhibiting HIF (FIH), regulates its transcriptional activity (Webb et al., 2009). FIH hydroxylates an asparginyl residue (803) preventing the interaction of HIF-α and transcriptional co-factors such as p300 and CREB binding protein, leading to its decreased transcriptional activity (Kaelin and Ratcliffe, 2008) (Figure 2A).

When oxygen availability is reduced the PHDs and FIH are inactive leading to the stabilization of HIF-α and its translocation into the nucleus. Once in the nucleus the HIF-α and HIF-1β subunits interact and bind to a hypoxia response element in the DNA resulting in the activation of target genes (Figure 2B). These genes facilitate adaptation and survival of cells and include genes involved in; angiogenesis, apoptosis, cell survival iron metabolism, inflammation, and metabolism (Kaelin and Ratcliffe, 2008).

#### Oxygen Independent

While VHL has been established as the key player in the oxygen-dependent degradation of HIF-α, a number of alternative regulatory pathways have been described including; receptor for activated C kinase 1 (Liu et al., 2007), hypoxia associated factor (Koh et al., 2008), integration site 6 gene (Chen et al., 2007).

HIF-α is also stabilized through oxygen-independent mechanisms via growth factors, cytokines, metabolites, oncogenes, tumor suppressor genes, nitric oxide, transition metals, and mechanical stress (reviewed Chun et al., 2002; Agani and Jiang, 2013). Furthermore, accumulating evidence has shown that HIF-α is activated by microorganisms, infection, and bacterial components (Peyssonnaux et al., 2005, 2007; Haeberle et al., 2008; Hartmann et al., 2008). However, the precise mechanisms involved remain to be fully elucidated and will be discussed in more detail below.

### HIF in Sepsis

Dynamic changes in HIF expression occur during sepsis, which has significant effects on cytokine production, metabolism, cell adaptation, and clinical symptomology. Therefore, studies have proposed HIF as a potential biomarker for sepsis, although this remains controversial. HIF-1 mRNA extracted from whole blood samples has been shown to be significantly higher in patients with septic shock. However, it did not correlate with patient outcomes (Textoris et al., 2012). In contrast, leukocyte HIF-1 mRNA and protein were found to be decreased in septic patients and inversely correlated with illness severity (Schäfer et al., 2013). Functionally active genetic variants in HIF-1 and PHD2 can impact on HIF-1α mRNA expression. However, they are not independent risk factors for 30-days mortality in severe sepsis (Höcker et al., 2015). A clinical trial investigating the potential of HIF-1 as a novel biomarker in septic shock has now been completed and data on the primary outcomes remains to be published (NCT02163473). The use of HIF as a biomarker may prove complex given its differential role depending on cell type. Furthermore, HIF can have different response depending on the type of bacteria causing the primary infection.

HIF has also been proposed as a potential therapeutic target for sepsis. However, to date studies have focused on indirect targeting of HIF. Edaravone, a potent radical scavenger, has been shown to induce HIF-1α, which in turn suppressed oxidative stress and protected the heart against septic myocardial injury and dysfunction (He et al., 2018). In contrast, 5,7-dihydroxy-8-methoxyflavone protected against sepsis-induced acute lung injury in part due to inhibition of HIF-1α accumulation (Sun et al., 2015). Similarly, co-stimulation of mice with LPS and the statin, simvastatin, decreased HIF-1α levels and protected against liver dysfunction in the early stage of sepsis (Yorulmaz et al., 2015). Further studies using specific HIF inhibitors will be required to fully elucidate the potential of this signaling pathway as a therapeutic target in sepsis.

### HIF, Innate Immune Cells, and Sepsis

Studies of LPS challenge in HIF-1 myeloid conditional knockout mice revealed that HIF-1 is a critical determinant of the sepsis phenotype, via the production of pro-inflammatory cytokines resulting in the clinical manifestation of sepsis symptomology including; tachycardia, hypotension, and hypothermia (Peyssonnaux et al., 2007; Fitzpatrick et al., 2018). Furthermore, inoculation of live or heat-inactive gram-positive bacteria into macrophages induced HIF-1α, while mice deficient in myeloid HIF-1, were shown to be protected against gram-positive endotoxin-induced sepsis mortality and clinical symptomology (Mahabeleshwar et al., 2011). Loss of PHD3, the enzyme responsible for the degradation of HIF, significantly shortened the survival of mice with abdominal sepsis due to an overwhelming innate immune response. Increased pro-inflammatory activity, in these mice, correlated with enhanced HIF-1 protein stabilization in macrophages (Kiss et al., 2012). Collectively, this data shows that HIF-1α contributes to the pathogenic role of macrophages in sepsis pathology. Additionally, pharmacological inhibition of HIF-1α using 2-Methoxyestradiol, protected mice from LPS and CLP induced sepsis. Suppressed HIF-1α induced iNOS/NO and cytokine production was found in the peritoneal macrophages, suggesting an important role of myeloid HIF-1α in sepsis survival (Yeh et al., 2011).

Additionally, HIF-2 also contributes to sepsis pathophysiology. HIF-2 myeloid conditional knockout mice with a deficiency in both macrophage and neutrophil HIF-2α, are protected, at least in part, against LPS endotoxemia-induced cardiac impairment and hypothermia and have enhanced overall survival rates in an LPS-induced endotoxemia model. These is due in part to an altered inflammatory response with mice expressing lower levels of pro-inflammatory cytokines but increased levels of anti-inflammatory cytokine (Imtiyaz et al., 2010).

In human blood monocytes isolated from patients, upregulated HIF1A and suppressed HIF2A expression was observed during gram-negative sepsis. However, this increase in HIF1A was not observed in the blood monocytes of these patients during the resolution phase (Shalova et al., 2015). Unlike, the conditional knockout studies discussed above, HIF-1α was shown to induce an endotoxin tolerant state, in these cells. The authors propose that an initial HIF-1α activation induces a pro-inflammatory phenotype. Whereas, chronic activation during sepsis, induces negative regulators of the inflammatory response thus dampening it. In support of this, they demonstrated that treatment of blood monocytes isolated from healthy donors with Lipid A (a component of gram negative bacteria) caused a HIF-1α induced upregulation of IRAKM, a negative regulator of TLR4. Furthermore, using siRNA they subsequently showed that this was responsible for downregulating pro-inflammatory cytokines such as TNF and IL-6 (Shalova et al., 2015).

Recently, we have shown that the role of myeloid HIF-1 in sepsis pathophysiology is time dependent. We found that mice with a specific myeloid HIF-1 deletion were initially protected against sepsis induced tachycardia, hypotension and hypothermia. However, at later time points myeloid HIF-1 had no effect on the clinical symptomology (Fitzpatrick et al., 2018). Taken together, these studies may suggest that HIF-1α has important temporal roles during sepsis; regulating the inflammatory response during the acute phase and regulating protective responses during the later stages. However, further studies will be required to shed more light on the different roles and time dependent aspects of myeloid HIF signaling during sepsis. In addition, the contribution of HIF activation in other innate immune cells, to sepsis pathophysiology, remains to be elucidated.

### HIF, Immunometabolism, and Sepsis

HIF is a major regulator of energy homeostasis in response to both hypoxic and non-hypoxic stimuli (Peyssonnaux et al., 2005, 2007; Kaelin and Ratcliffe, 2008). In sepsis, the majority of studies have focused on HIF-dependent glycolysis. Evidence is emerging for a role of HIF in regulating other metabolic pathways. However, our understanding is complicated by the fact that HIF appears to have cell specific roles in controlling metabolism in different immune cells (Table 1).

**Table 1:** Summary of the metabolic pathways regulated by HIF in innate immune cell subpopulations during sepsis.

**Cell type**	**Early metabolic response**	**Late metabolic response**
Macrophage	Activated HIF-la: ↑Giycolysis ↑Nitric oxide production ↑Pentose phosphate pathway ↓Oxidative phosphorylation Suppressed HIF-2a: ↓Arginase activity	Suppressed HIF-la: ↑Fatty acid oxidation
Dendritic cell	Unknown	Activated HIF-la: ↑Glycolysis
Neutrophil	Activated HIF-la: ↑Glycolcysis	Unknown

#### Glycolysis

Previous studies have shown that the molecular mechanism underlying the switch from OXPHOS to glycolysis during innate immune cell response to sepsis requires HIF-1α (Liu et al., 2012; Tannahill et al., 2013; Meiser et al., 2015; Wang et al., 2017). Indeed, in macrophages HIF-1α up-regulates key components of the glycolytic pathway including; the glucose transporter Glut1, phosphofructose kinase, hexokinase, phosphoglucomutase-2, enolase 2, lactate dehydrogenase, and pyruvate dehydrogenase kinase 1 (Liu et al., 2012; Tannahill et al., 2013; Piñeros Alvarez et al., 2017). While depletion of HIF-1α expression reduces basal glucose uptake and glucose oxidation and prevents against the activation of the glycolytic pathway (Liu et al., 2012). Interestingly, in a whole animal model of LPS induced endotoxemia, we have recently shown that myeloid HIF-1 plays an acute time dependent role in regulating the peripheral glycolytic response (Fitzpatrick et al., 2018). We observed that mice with a specific deletion of myeloid HIF-1 had a time-dependent shift in the development of hypoglycaemia and glucose uptake into the heart and brown fat, which correlated with a protective effect on cardiac dysfunction and body temperature dysregulation (Fitzpatrick et al., 2018). Similarly, in human dendritic cells the increased glucose consumption and transcription of hexokinase-2, triggered by LPS is inhibited following pharmacological inhibition of HIF-1α (Perrin-Cocon et al., 2018). Intriguingly, in mouse dendritic cells HIF-1α is required for the later shift to glycolysis but the immediate metabolic switch is HIF-1α independent (Jantsch J et al., 2008). While in neutrophils LPS stimulation did not cause an increase in Glut1 expression it did cause the translocation of Glut1 to the cell surface, due to HIF-1α activation (Schuster et al., 2007). In contrast, myeloid HIF-2α deficiency does not altered the glycolytic response to sepsis (Imtiyaz et al., 2010).

#### Arginine Metabolism

HIF-1α activation is not limited to the regulation of glycolysis. Interestingly, HIF1α and HIF2α exert opposing effects on arginine metabolism in response to LPS in macrophages. HIF1α is induced leading to enhanced iNOS expression and NO production, while HIF2α is suppressed resulting in decreased arginase activity (Takeda et al., 2010). These findings were further validated in a mouse model of LPS-induced endotoxemia. Plasma levels of NO were reduced in myeloid specific HIF-1α knockout mice but increase in myeloid-specific HIF-2α knockout mice (Takeda et al., 2010).

#### Other Metabolic Pathways

While data shows a clear role for HIF in regulating glycolysis and arginine metabolism in innate immune cells during sepsis, its contribution the regulation of other metabolic pathways remains to be determined. Evidence shows that under different conditions HIF can regulate additional metabolic pathways, thus it is plausible that HIF will have an important role in controlling these metabolic processes in sepsis. For example, in addition to glycolysis, glucose is also metabolized through the PPP. Recently, it has been shown that key components of the PPP pathway including; fructose 1,6-bisphosphate, fructose-6-phosphate, glyceraldehyde-3-phosphate, glucose-6-phosphate, phosphoenolpyruvate, 3-phosphoglycerate are increased in macrophages overexpressing HIF-1α (Wang et al., 2017).

Pyruvate the end product of glycolysis enters the TCA cycle only via the production of acetyl-CoA. In the presence of low oxygen HIF-1 suppresses metabolism through the TCA cycle by directly trans-activating the gene encoding pyruvate dehydrogenase kinase 1 (PDK1), that in turn inactivates the TCA cycle enzyme, pyruvate dehydrogenase (PDH), which converts pyruvate to acetyl-CoA (Kim et al., 2006). Interestingly, however, in sepsis evidence to date has shown that TCA intermediates regulate HIF expression during metabolic reprogramming (Tannahill et al., 2013).

The role of the HIF signaling pathway in regulating fatty acid oxidation has to date primarily been explored within the liver, where both HIF-1α and HIF-2α have been found to play important roles (Rankin et al., 2009; Qu et al., 2011; Huang et al., 2014; Liu et al., 2014; Ramakrishnan and Shah, 2017; Arai et al., 2018). Future research will determine the contribution that both isoforms make to the complex changes observed in fatty acid oxidation during sepsis. Given that HIF-1α and HIF-2α have specific and different temporal and functional roles (reviewed Koh and Powis, 2012), it will be interesting to determine if the switch from glycolysis in the acute phase of sepsis to fatty acid oxidation in the later phase is regulated by a switch from HIF-1α to HIF-2α signaling. Indeed, in TLR4 stimulated macrophages, a previous study showed that HIF-1α drives the early glycolytic response but is suppressed during the late phase allowing increased fatty acid β oxidation, however the role of HIF-2α was not explored (Liu et al., 2012).

### Molecular Mechanisms Driving HIF Immunometabolism During Sepsis

While we now have strong evidence to support HIF induced glycolytic reprogramming of innate immune cells during sepsis, the molecular mechanisms leading to HIF stabilization are not as well-characterized. Further complexity exists as the intracellular pathways involved in non-Hypoxic HIF activation seem to be cell and stimulus specific. Thus, multiple molecular pathways likely play important roles in the HIF-mediated metabolic reprogramming of innate immune cells during sepsis.

#### TLR's

TLR's belong to a larger family of receptors called pattern-recognition receptors, which are expressed on cells of the innate and adaptive immune cells. They play a vital role in pathogen recognition and innate immunity. To date 10 distinct TLR's have been identified in humans, each of which recognizes a different microbial motif (Pandey et al., 2014). Activation of HIF-1α by TLR's is essential in the metabolic reprogramming of innate immune cells during sepsis (Perrin-Cocon et al., 2018). Early studies found that TLR4 can increase HIF-1α accumulation by decreasing its degradation. In macrophages LPS induced TLR4 stimulation decreased the mRNA levels of the PHD2 and 3 enzymes (Peyssonnaux et al., 2007). More recently, in dendritic cells TLR4 driven glycolysis has been found to correlate with HIF-1α stabilization (Perrin-Cocon et al., 2018). Interestingly, while activation of the TLR1/2 and TLR2/6 receptors also stimulates glycolysis in dendritic cells, it appears to be through a HIF-1α independent mechanism (Perrin-Cocon et al., 2018).

Due to the potent harmful effects of TLR signaling, humans have evolved numerous mechanisms to enable us to dampen and protect against aberrant activation. Suppressor of cytokine signaling 1 (SOCS1) is one negative regulator of TLR signaling and thus innate immunity. Pharmacological inhibition of SOCS1 and myeloid cell-specific deletion of SOCS1 increases the susceptibility to mice to sepsis (Kinjyo et al., 2002; Nakagawa et al., 2002; Piñeros Alvarez et al., 2017). This is accompanied by metabolic reprogramming of the macrophage as evident by increased lactic acid production and elevated expression of glycolytic genes such as hexokinase, lactate acid dehydrogenase A and Glut1 in septic mutant mice. This upregulation was shown to be due to an increase in HIF-1α driven glycolytic reprogramming of macrophages (Piñeros Alvarez et al., 2017).

#### Transcription Factors

Downstream signaling of the TLR's leads to the activation of a wide range of transcription factors which promote the stabilization of HIF-1α during sepsis, including; mitogen-activated protein kinase (MAPK), MAPK phosphatases-1 and NFκB (Frede et al., 2006; Rius et al., 2008; Talwar et al., 2017).

NFκB serves as a master regulator of the immune response though the regulation of multiple aspects of immune cell function (reviewed Lawrence, 2009). It is the collective name for a group of inducible transcription factors that exist as homo- or hetero-dimers of the following subunits; RelA (p65), RelB, cRel, p50, and p52. In the inactive state NFκB proteins are sequestered in the cytoplasm by a class of inhibitory proteins known as inhibitors of NFκB (IκB). The activation of NFκB involves two major signaling pathways, the canonical and non-canonical pathways (Lawrence, 2009). The canonical pathway is stimulated by a variety of factors including TRL and microorganisms. These pathways converge on a complex of three proteins known as IKK. This complex consists of two catalytic subunits (IKKα and IKKβ) and a regulatory subunit (IKKγ). IKK phosphorylates IκB, which in turn triggers the proteasomal degradation of IκB and the rapid nuclear translocation of canonical NFκB members (Taylor and Cummins, 2009). NFκB regulates LPS enhanced transcription of HIF-1α in human monocytes and macrophages by binding to a response element present in the HIF-1α promoter (Frede et al., 2006). These findings were recapitulated in an *in vivo* model of sepsis. In macrophages lacking IKKβ, NFκB was demonstrated to be a vital transcriptional activator of HIF-1α. Furthermore, IKKβ deficiency resulted in suppressed induction of various HIF-1α metabolic genes including Glut1, PGK and iNOS (Rius et al., 2008). The molecular mechanism through which NFκB regulates HIF-1α have been further explored in dendritic cells. LPS induced NFκB upregulated the iron storage protein ferritin, reducing the free iron availability. Low iron levels in turn impaired the PHD enzyme activity, thus allowing HIF-1α stabilization (Siegert et al., 2015). In contrast other studies have shown that LPS mediated activation of HIF is independent of NFκB (Nishi et al., 2008).

MAPK are a highly conserved family of serine/threonine kinases that regulate a wide range of cellular processes including gene expression and metabolism (reviewed Cargnello and Roux, 2011). In mammals, 14 MAPKs have been identified of which the ERK1/2 (p42/44), JNKs and p38 isoforms are the most extensively studied (Cargnello and Roux, 2011). In macrophages and monocytes, LPS induced HIF-1α accumulation was demonstrated to involve p44/p42 MAPK (Frede et al., 2006). While, in dendritic cells the LPS driven TLR4 glycolytic burst requires p38-MAPK-dependent HIF-1α accumulation and transcriptional activity (Perrin-Cocon et al., 2018). The activation of MAPK is negatively regulated by MAPK phosphatases (MKPs). Macrophages deficient in MKP respond to LPS challenge by decreasing levels of all three PHD enzymes and increasing p38 MAPK activity both leading to increased stability of HIF-1α and NO production (Talwar et al., 2017).

The interferon regulatory factors (IRFs), that consist of nine members in mammals, act as transcription factors for interferons and thus play numerous roles in host response to infection (Zhang et al., 2015b). IRF2 has distinct role in regulating glycolysis during sepsis (Cui et al., 2018). It undergoes early proteasomal degradation in LPS treated macrophages leading to HIF-1α glycolytic gene expression and thus cellular glycolysis. IRF2 regulates HIF-1α driven glycolysis at the transcriptional level with enhanced HIF-1α binding to the promoter regions of glycolytic genes found in IRF2 knockout macrophages (Cui et al., 2018).

#### Metabolic Pathways and By-Products

It has recently emerged that by-products of metabolism also regulate HIF activity in sepsis. For example, pyruvate kinase M2 (PKM2) is the rate limiting enzyme of glycolysis and is a critical determinant of the metabolic reprogramming of macrophages via HIF in response to LPS. In primary macrophages, PKM2 forms a complex with HIF-1α in the nucleus, which enhances glycolysis by inducing the expression of glycolytic enzymes including hexokinase 2 and lactate dehydrogenase A (Yang et al., 2014; Palsson-McDermott et al., 2015). Moreover, both HIF-1α expression and glycolytic rates are dramatically reduced in PKM2 deficient macrophages (Palsson-McDermott et al., 2015). Interestingly, under these conditions the essential fatty acid, ω-Alkynyl arachidonic acid, can disrupt the interaction between PKM2 and HIF-1α. Macrophages pre-treated with ω-Alkynyl arachidonic acid had decreased PKM2 expression and nuclear translocation following LPS stimulation. This markedly reduced the formation of PKM2-HIF-1α complexes, attenuating the binding of HIF-1α to the iNOS promoter, thus decreasing the expression of iNOS. Increase arginase 1 was also observed in these cells suggesting that arginine metabolism maybe altered in LPS-stimulated macrophages in the presence of ω-Alkynyl arachidonic acid (Cheng et al., 2017).

Succinate is an important intermediate of the TCA cycle and plays a key role in ATP generation in the mitochondria. Emerging evidence has identified roles for succinate outside metabolism including stabilization of HIF-1α in LPS activated macrophages. The accumulation of succinate under these conditions suppresses the activity of the PHD enzymes and induces ROS resulting in the stabilization of HIF-1α and the subsequent induction of glycolysis (Tannahill et al., 2013). In addition, succinate has been shown to be partially derived from glutamine via the GABA shunt and inhibition of this pathway lowers succinate levels and in turn decreases HIF-1α stabilization in murine macrophages and sepsis (Tannahill et al., 2013). Itaconate a metabolite generated by the mitochondrial enzyme, immune responsive gene 1 (Irg1), regulates succinate levels. It functions by inhibiting succinate dehydrogenase (Sdh) mediated oxidation of succinate to fumarate, thus allowing succinate to accumulate. Interestingly, following LPS stimulation Irg1 deficient macrophages decreased succinate accumulation yet increased fumarate and malate concentrations indicative of Sdh activity. Furthermore, lack of Irg1 increased oxygen consumption rates and HIF-1α mRNA and protein and NO levels (Lampropoulou et al., 2016). Therefore, changes in HIF-1α correlate with the efficiency of succinate oxidation by Sdh. This led the authors to propose that HIF-1α is linked to the efficiency and directionality of the electron transport chain in LPS stimulated macrophages as oppose to direct signaling through succinate accumulation (Lampropoulou et al., 2016).

Metabolic by products also suppress HIF-induced metabolic reprogramming during sepsis. The aromatic ketoacids, indolepyruvate, decreases LPS induced glycolysis, the glycolytic capacity and the glycolytic reserve in macrophages. An effect due to increased HIF-1α hydroxylation and degradation, which in turn suppresses the expression of key glycolytic genes; Glut1 and lactate dehydrogenase A (McGettrick et al., 2016). Similarly, when the omega-7 monounsaturated fatty acid, palmitoleic acid (PM), was co-incubated with LPS, PM reduced the expression of TLR4 and HIF-1α promoting reduced glycolysis and an anti-inflammatory effect in macrophages (Souza et al., 2017). Furthermore, α-ketoglutarate (α-KG) produced during the TCA cycle, also suppresses HIF-1α through increased PHD enzyme activity (Tannahill et al., 2013). Finally, glycolysis can also regulate HIF-1α. The glycolytic inhibitor 2 deoxy-D-glucose suppresses HIF-1α in activated macrophages (Tannahill et al., 2013; Yang et al., 2014).

#### Kinases

Kinases are highly conserved in evolution and represent one of the largest gene families in eukaryotes (Rauch et al., 2011). They play a role in diverse signal transduction pathways that modulate a range of cellular processes including metabolism and have been proposed has both biomarkers and therapeutic targets for sepsis (Schenck et al., 2019). Emerging data has identified numerous kinases in regulating HIF- dependent metabolism in response to sepsis.

Mammalian target of rapamycin (mTOR) is an evolutionary conserved serine/threonine kinase that acts as a master regulator of cellular metabolism. Studies have demonstrated that LPS stimulation of innate immune cells mediated HIF-1α activation via mTOR (Zhang et al., 2015; Lopez-Pascual et al., 2018). Furthermore, mTOR-dependent HIF-1α dependent metabolism is highly active in leukocytes in both *E. coli* and *Candida sepsis* (Cheng et al., 2016) More recently the upstream signaling mechanisms leading to mTOR activation have been elucidated. In LPS stimulated human and mouse neutrophils it was shown that upon engagement of TRL4, PI3K is activated and promotes matrix metallopeptidase 9 expression, and AMPKα cleavage. This enables mTOR activation, which induces HIF-1α expression (Zhang et al., 2015).

The serine/threonine kinase AMP-activated protein kinase (AMPK) is a central regulator of energy homeostasis and thus coordinates multiple metabolic pathways (Kim et al., 2016). AMPK is a negative regulator of metabolic reprogramming of immune cells and thus suppresses sepsis development *in vivo* (Huang et al., 2018). Pharmacological activation of AMPK in macrophages results in diminished LPS-induced HIF-1α accumulation, which in turn diminishes the severity of lung injury following polymicrobial sepsis (Liu et al., 2015).

Shingosine kinase 1 (SphK1) is a vital for controlling the cellular response to various stress stimuli (Pchejetski et al., 2010). In macrophages shingosine kinase 1 (SphK1) mediates LPS-induced, ROS-dependent activation of HIF-1α through extracellular signal-regulating kinase, PLC-1γ and PI3 kinase pathways. SphK1 then contributes to the assembly of the NADPH oxidase complex that produces ROS and activates HIF-1α (Pchejetski et al., 2010).

#### ROS

ROS, generated in both the cytosol and mitochondria, modulate various physiological processes including metabolism and act as key signaling molecules (Ohl and Tenbrock, 2018). During sepsis, the generation of ROS requires the activation of TLR4 and myeloid differentiation factor (MyD)88 (Nishi et al., 2008; Liu et al., 2012). ROS subsequently activate HIF-1α, an effect that is blocked by treatment with antioxidants and a NADPH oxidase inhibitor (Nishi et al., 2008). LPS activated TLR4 has also been shown to regulate ROS production and HIF-1α expression via the p38 MAPK pathway. Pre-treatment of BMDM's with a p38 MAPK specific inhibitor decreased the LPS mediated production of both cytosolic and mitochondrial ROS and HIF-1α (Talwar et al., 2017).

#### Epigenetic Modifications

Using an *in vitro* model of sepsis the mechanism underlying the switch from HIF-1α dependent glycolysis in the early phase of sepsis to PGC-1 dependent fatty acid β oxidation during the late response to sepsis has been explored in macrophages. This switch requires nicotinamide phosphoryltransferase (Nampt) dependent generation of NAD^+^, which was sensed by the deacetylase sirtuin 1 (SirT1) and SirT6. SirT6 reduced glycolysis by epigenetically silencing HIF-1α and SirT1 directly binds and activates PGC-1 coactivators to support fatty acid β oxidation (Liu et al., 2012).

#### Other

Amyloid beta A4 precursor protein-binding family A member 3 (APBA3) is a protein coding gene, that enhances the activity of HIF-1α in macrophages by suppressing the activity of FIH (Sakamoto and Seiki, 2009). A previous study demonstrated that knockdown of APBA3 in macrophages redistributes FIH to the cytoplasm, suppressing HIF-1α dependent glycolysis and ATP production (Sakamoto and Seiki, 2009). A subsequent study showed that both APBA3-deficient mice and mice with a conditional knockout of the Apba3 gene in cells of the myeloid lineage are resistant to LPS-induced septic shock due to decreased glycolysis and cytokine production. Macrophages from APBA3 knockout mice showed a 40% reduction in ATP and decreased glycolytic activity due to a reduction in the expression of HIF-1α dependent glycolytic genes including; Glut1 and PGK-1 (Hara et al., 2011).

## Conclusion and Future Prospective

**Figure 3:**
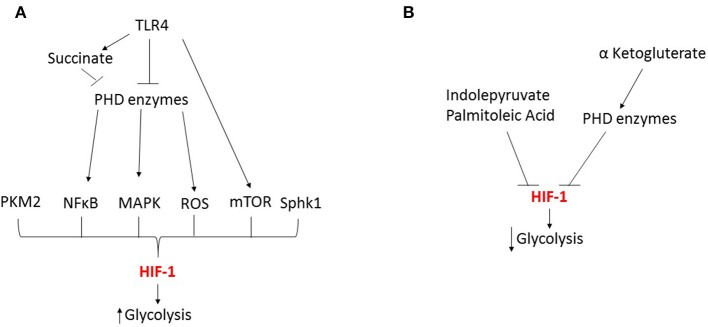
HIF-1 dependent regulation of glycolysis during sepsis. **(A)** During the early response to sepsis TRL4 activation inhibits the prolyl hydroxylase (PHD) enzymes leading to the activation of transcription factors NFκB and MAPK, kinases including mTOR, reactive oxygen species (ROS) and metabolic by-products such as succinate and pyruvate kinase M2 (PKM2) leading to HIF activation and enhanced glycolysis. **(B)** Metabolic by-products can inhibit HIF leading to reduced glycolysis.

During sepsis, evidence shows that HIF is critically involved in mediating numerous metabolic changes in innate immune cells during both the hyperinflammatory and immunotolerant phases. However, HIF's role in mediating these changes is complex and several key issues remain to be addressed. In particular, the dynamics of the HIF-1α and HIF-2α responses differ and this has significant effects on the timing of the metabolic changes. In addition, to date most investigations have focused on the role of HIF in regulating glycolytic changes (Figure 3), but HIF's role in controlling other metabolic pathways is much less characterized. Therefore, future studies looking at the time dependent effects of both HIF isoforms on innate immune cell metabolism during each phases of sepsis will provide a clearer understanding of the role of HIF in metabolic reprogramming. However, this will be complicated by numerous factors including; differences between innate immune cell subpopulations and the underlying pathogen.

Sepsis involves multiple innate immune cells: neutrophils, dendritic cells, macrophages, monocytes, and natural killer cells. Studies in tumor models have shown that HIF can have different roles in immune cell subpopulations under the same conditions (Doedens et al., 2010; Kim et al., 2012). In addition, targeted deletion of HIF in one cell type can have significant effects of the recruitment, activation and function of other cell types. To date mechanistic studies in sepsis have looked at the role of HIF in a single cell type. However, *in vivo* the situation is significantly more complex due to the interplay between innate immune cell subpopulations and innate immune cells and those of the adaptive immune system and the local stromal environment. In depth understanding of the metabolic pathways controlled by HIF in each subpopulation of innate immune cells and how the interconnections between cell types effect metabolic change will be vital in unraveling the complex HIF dependent metabolic changes during sepsis.

The molecular mechanisms governing HIF stabilization during sepsis are complex. However, there is still much to be discovered regarding the mechanistic regulation of HIF. Furthermore, our understanding of how HIF induces metabolic changes is also limited at present. It is likely that HIF through diverse mechanisms such as, activation of molecular signaling pathways, post-translational modification, and epigenetic alterations, tightly governs metabolic alterations. Understanding these mechanisms should identify new avenues for the development of novel strategies for sepsis.

One final limitation of current studies to consider is that most studies have used LPS-induced endotoxemia as a model of sepsis. While this model can re-capitulate many of the clinical symptoms associated with sepsis, at the molecular level important differences remain. Due to the complex and heterogenous nature of the condition a perfect animal model is untenable. Therefore, studies using human cells from patients and multiple different animal models including pathogen-based models will provide a more clinically relevant understanding of the metabolic alterations which occur in innate immune cells and the role of HIF signaling. Addressing these current issues/limitations will be vital in order to determine the validity of HIF as a diagnostic and therapeutic target for sepsis.

## Author Contributions

The author confirms being the sole contributor of this work and has approved it for publication.

### Conflict of Interest Statement

The author declares that the research was conducted in the absence of any commercial or financial relationships that could be construed as a potential conflict of interest.
